# Boredom proneness and inattention in children with and without ADHD: the mediating role of delay aversion

**DOI:** 10.3389/fpsyt.2025.1526089

**Published:** 2025-04-28

**Authors:** Chia-Fen Hsu, Vincent Chin-Hung Chen, Hsing-Chang Ni, Ning Chueh, John D. Eastwood

**Affiliations:** ^1^ Department of Occupational Therapy, College of Medicine, National Cheng Kung University, Tainan, Taiwan; ^2^ School of Medicine, Chang Gung University, Taoyuan, Taiwan; ^3^ Department of Psychiatry, Chang Gung Memorial Hospital, Chiayi, Taiwan; ^4^ Department of Psychiatry, Chang Gung Memorial Hospital at Linkou, Taoyuan, Taiwan; ^5^ Department of Psychiatry, National Taiwan University Hospital, Hsin-Chu, Taiwan; ^6^ Department of Psychology, Faculty of Health, York University, Toronto, ON, Canada

**Keywords:** ADHD, boredom, delay aversion, mediator, inattention

## Abstract

**Objective:**

A high tendency to boredom and delay aversion are commonly observed traits among children and adolescents diagnosed with ADHD. However, the interplay between boredom, delay aversion, and ADHD symptoms remains unclear. It is unknown, for example, whether a predisposition to boredom predicts difficulties in sustaining attention because of susceptibility to delay aversion. This study investigated the potential mediating role of delay aversion in the relationship between boredom and inattentive behaviors in children with and without ADHD.

**Methods:**

Parent- and self-ratings of boredom proneness, delay aversion, and inattention symptom severity of 93 children with ADHD and 90 typically developing controls (aged 9–16 years) were included in analysis.

**Results:**

Both parent- and self-ratings showed that children with ADHD had significantly elevated levels of trait boredom, delay aversion, and inattention compared to controls. Trait boredom, delay aversion, and inattention were inter-correlated. The parent-ratings showed that children with a higher level of trait boredom tended to be more inattentive, and this effect was partially explained by the mediating role of delay aversion.

**Conclusions:**

Our findings suggest that the relation between boredom and inattention may be at least partially explained by the mediating role of delay aversion in children within and without ADHD.

## Highlights

- Children with ADHD are more prone to boredom than healthy controls.
- Children with ADHD are more delay aversive than healthy controls.
- Trait boredom, delay aversion, and ADHD inattentive behaviors were inter-correlated.
- Stable disposition of boredom predicts ADHD inattentive behaviors.
- Delay aversion partially mediates the association between trait boredom and ADHD inattentive behaviors.

## 1 Introduction

### 1.1 The concept of boredom

Boredom has become an increasing problem, with approximately 20% of adolescents reporting that they experience high levels of boredom (1). Cognitively, boredom proneness is closely related to problems with attention regulation (2, 3). Emotionally, boredom proneness is associated with frustration, particularly when individuals feel that they have little control or independence in task execution. Conversely, in situations of high task autonomy, boredom may trigger feelings of depression (4). Clinically, boredom proneness is associated with an increased risk of problematic eating behavior (5), substance use (6), problem gambling (7), and attention deficit/hyperactivity disorder (ADHD)-like symptoms (8, 9).

### 1.2 Boredom, ADHD, and inattention

ADHD is one of the most common neurodevelopmental disorders of childhood, affecting 5.3%–7.1% of children and adolescents worldwide (10). Research has shown that parent-assessed boredom proneness scores for children with ADHD were higher than those for typically developing controls, and children with ADHD exhibited higher levels of state boredom after completing a continuous performance task (11). Additionally, children with ADHD engage in more inappropriate behaviors during idle time in the classroom than typically developing controls (12). Recently, during the COVID-19 pandemic, boredom was reported as one of the top problems among adolescents and young adults with ADHD (13). The elevated proneness to boredom in children with ADHD appears to be improved by medication (14). Notably, boredom has implicated the brain’s default mode network, where individuals with ADHD exhibit neural abnormalities during resting, goal-directed behavior, and waiting (15–17).

Studies with non-clinical, university student samples have also suggested that proneness to boredom is positively associated with inattentive behaviors (9, 18), impulsiveness (19, 20), attentional lapses in everyday activities (9), and performance errors on tasks requiring sustained attention (9, 18, 21). Thus, taken together, existing research suggests a clear association between trait boredom and ADHD symptoms as well as an association between trait boredom and inattention in non-clinical samples.

### 1.3 Delay aversion in ADHD

Difficulty waiting is also a common problem for children with ADHD. The delay aversion hypothesis suggests that symptoms of ADHD reflect alterations in the brain’s dopaminergic reward system, whereby the value of delayed rewards is discounted to a greater extent than normal (22). Individuals with ADHD often face adverse consequences when confronted with delays. Over time, they develop a pervasive aversion to delays. Therefore, they typically opt for immediate or less-delayed options over larger, delayed ones, a phenomenon termed choice impulsivity. When delay is not possible to avoid, individuals with ADHD express maladaptive behaviors such as distraction, inattention, or overactivity, possibly for the purpose of decreasing the perception of waiting (23).

Converging evidence supports the idea that individuals with ADHD tend to make impulsive choices when faced with a delay (24). Neuroimaging studies found a delay-related increase of neural activity in the brain’s emotion areas in individuals with ADHD compared with healthy controls, suggesting that delay led to unpleasant emotions and feelings to ADHD (25), and this relationship seems to be mediated by activation in the amygdala and dorsolateral prefrontal cortex (26).

One of the unpleasant feelings triggered by waiting is boredom—for example, a recent study that assessed people’s experience of boredom and subjective time during real waiting situations found that state boredom and more frequent thoughts about time resulted from having to wait (27). Importantly, environmental stimulation during waiting has been shown to effectively reduce restlessness and inattention (23), which may suggest that it is not waiting per se but the feeling of boredom during a wait that is critically related to inattentive behaviors.

### 1.4 Goals of the current study

Although previous research has established a robust association between boredom proneness and ADHD symptoms as well as between boredom proneness and inattention in non-clinical samples, the psychological mechanism which links boredom proneness and inattention is unknown. Moreover, although waiting is associated with boredom, boredom proneness has not been examined as a potential contributor to delay aversion. In the present work, we seek to explore the possibility that boredom proneness predicts increased delay aversion, which, in turn, predicts inattention in children with and without ADHD—that is, we examine the potential role of delay aversion in mediating the relation between boredom proneness and inattention.

## 2 Materials and methods

### 2.1 Ethics statement

The experimental protocol was approved by the local ethics committee (Chang Gung Medical Foundation Institutional Review Board, IRB No. 201701995B0). This study was performed in accordance with the Declaration of Helsinki and its later amendments. Written informed consent was obtained from all child participants and their parents or guardians before data collection.

### 2.2 Subjects

A total of 183 children with and without ADHD (aged 9 to 16 years) participated in this study. The participants were recruited from Chang Gung Memorial Hospital (Chiayi and Linkou branches) and local communities. Of the 183 children, 93 had a diagnosis of ADHD. ADHD diagnosis was confirmed by senior child psychiatrists based on the Diagnostic and Statistical Manual of Mental Disorders, Fifth Edition (DSM-V). The exclusion criteria were (a) the presence of neurodevelopmental or psychiatric disorders other than ADD/ADHD and (b) estimated IQ less than 70. The children completed four subtests of the Wechsler Intelligence Scale for Children, 4th Edition (WISC-IV), to estimate their IQ (28) and self-rating scales of boredom and delay aversion. Their parents or primary caregivers completed the parent-rating scales of boredom, delay aversion, and ADHD symptom severity.

### 2.3 Short Boredom Proneness Scale

The Short Boredom Proneness Scale (SBPS; 29) is a self-rating instrument that contains eight items to assess trait boredom. The items were translated into traditional Chinese with culturally relevant colloquial expressions by a board-certificated psychologist in Taiwan (the first author). We produced a self-report and a parent report by replacing the subject of the statement (I to he/she). The statements were then back-translated independently by an expert bilingual panel. The back-translation was provided to the leading author of the SBPS (Dr. Danckert), who identified inadequate expressions. This approach was repeated until all inadequate expressions in the translation were resolved. The translated scale used a five-point Likert scale ranging from 1 (strongly disagree) to 5 (strongly agree) because the original SBPS study found that participants can have difficulty in differentiating the response options on a seven-point Likert scale (29). The responses were summed to derive a total score, with higher scores reflecting a higher proneness to boredom. Confirmatory factor analysis (CFA) with the control sample for parent-rating indicated a good fit to the data: *X* ^2^/*df* = 22.77/20, RMSEA = .04, CFI = .99, TLI = .99, SRMR = .04. Cronbach’s alpha coefficient was.87. CFA with the control sample for the self-rating indicated an acceptable fit to the data: *X* ^2^/*df* = 33.45/20, RMSEA = .09, CFI = .89, TLI = .85, SRMR = .07. Cronbach’s alpha coefficient was.74.

### 2.4 Quick Delay Questionnaire

The Quick Delay Questionnaire (QDQ; 30) is a self-rating scale to assess delay-related feelings and behaviors in everyday life. The QDQ contains 10 items on a five-point Likert scale ranging from 1 (not like me at all) to 5 (very like me). The responses were summed to derive a total score, with higher scores reflecting greater delay aversion. The original QDQ was translated into traditional Chinese and the subject was replaced to create a parent-rating version. The items were back-translated independently by a bilingual expert panel following the procedure described above for the SBPS. The back-translation was approved by the leading author of the QDQ (Prof. Sonuga-Barke). In everyday life, we encounter situations where waiting may or may not be inevitable. To streamline the investigation, we employed the total score of QDQ to refer both choice impulsivity (when the option to evade delay exists) and the aversive feelings associated with forced delay (when there is no opportunity to escape waiting). CFA for the total score of parent-rating in the control sample indicated a good fit: *X* ^2^/*df* = 58.51/35, RMSEA = .09. CFI = .93, TLI = .92, SRMR = .07. Cronbach’s alpha was.76. CFA for the total score of self-rating in the control sample indicated that the model fit was acceptable: *X* ^2^/*df* = 68.18/35, RMSEA = .10, CFI = .77, TLI = .71, SRMR = .09. Cronbach’s alpha coefficient was.73.

### 2.5 Swanson, Nolan, and Pelham, version IV, parent-rating

The parents completed the Chinese version of the Swanson, Nolan, and Pelham scale, version IV (SNAP-IV) to assess the severity of their child’s ADHD symptoms. The SNAP-IV is a four-point Likert scale with established psychometric properties (31). Out of the 26 items in the SNAP-IV, nine items are parallel to the core inattention symptoms of ADHD (nine items), nine items are for hyperactivity/impulsivity symptoms, and eight items are parallel to oppositional defiant disorder based on the DSM-IV.

### 2.6 Statistical analysis

All scores were within three standard deviations of the mean with the whole group, except five data points from the SNAP-IV hyperactivity subscale. These data points were all rated by parents of children with ADHD and were not outliers relative to the ADHD group distribution; thus, the data of these subjects were retained in the analysis. We used independent-sample *t*-tests and chi-square tests to compare group differences according to the type of data. We applied correlational and multiple regression analyses to the variables of interest. We used SPSS macro PROCESS v3.4 (32) to examine the mediatory roles of delay aversion in the relation between boredom proneness and inattentive behaviors. The number of bootstrapping was 5,000. Since both our independent and dependent variables are measured within a single survey, we assessed common method bias (CMB) using Harman’s single factor score, in which all parent and self-rating measures were loaded onto a single common factor (33). The total variance explained by this factor is 42.45%, which is below than the 50% threshold, indicating that CMB does not affect the data.

## 3 Results

### 3.1 Descriptive statistics and group differences

Presented in **Table 1** are the demographics and clinical characteristics of the ADHD and control groups. There were no group differences in age, gender, or estimated IQ (*p* >.05). We asked the parents to select the category that best represents their children’s academic performance, and the ADHD group had a higher proportion of students in the lower academic performance category (*X* ^2^ = 33.99, *p* <.001). The ADHD group had more severe inattentive and hyperactive/impulsive behaviors than the control group on SNAP-IV (*t*(181) = −10.39, *p* <.001; *t*(213) = −7.79, *p* <.001). Both the parent- and self-reports of SBPS and QDQ showed that the ADHD group displayed a significantly elevated level of trait boredom (parent-rating: *t*(181) = -8.09, *p* <.001; self-rating: *t*(181) = −2.68, *p* = .01) and delay aversion (parent-rating: *t*(181) = -6.78, *p* <.001; self-rating: *t*(181) = −3.63, *p* <.001) compared to the control group.

**Table 1:** Demographics and clinical characteristics.

	Control (*n* = 90) Mean (SD)	ADHD (*n* = 93) Mean (SD)	*t*/*X* ^2^	*p*	95% CI
**Age**	12.33 (1.92)	11.97 (1.96)	1.28	.20	(-.20,.93)
**Gender (M/F)**	16 F/74 M	12 F/81 M	.84	.36	
**School performance (%)** **Excellent** **Above average** **Average** **Below average** **Lower tier**	17.8 41.1 31.1 5.6 4.4	8.6 19.4 23.7 30.1 18.3	33.99	<.001	
**Estimated IQ**	103.70 (12.04)	100.64 (13.41)	1.62	.11	(-.67, 6.78)
**SNAP-IV (parent-rating)**					
**Inattention**	7.18 (4.12)	15.17 (6.13)	-10.39	<.001	(-9.51, -6.48)
**Hyperactivity/impulsivity**	3.71 (3.32)	9.38 (6.15)	-7.79	<.001	(-7.10, -4.23)
**SBPS**					
**Parent-rating**	20.49 (6.40)	28.19 (6.49)	-8.09	<.001	(-9.59, -5.83)
**Self-rating**	21.49 (5.55)	23.94 (6.73)	-2.68	.01	(-4.24, -.65)
**QDQ**					
**Parent-rating**	26.13 (5.93)	32.86 (7.44)	-6.78	<.001	(-8.69, -4.77)
**Self-rating**	25.50 (6.30)	29.25 (7.59)	-3.63	<.001	(-5.79, -1.71)

SBPS, Short Boredom Proneness Scale; SNAP-IV, Swanson, Nolan, and Pelham, version IV; QDQ, Quick Delay Questionnaire; *t*/*X*², *t*-test or chi-square test statistic; CI, confidence interval.

### 3.2 Correlational and regression analyses

**Table 2** displays the correlation coefficients among SNAP-IV inattention score, parent- and self-ratings of SBPS and QDQ, controlling for age and gender. The analysis was conducted using the whole sample, as separate analysis for the ADHD group and control groups yielded similar results. The scores on inattention, boredom, and delay aversion were positively correlated. The correlations between inattention and the parent-ratings of SBPS and QDQ showed a strong effect size. The correlations between inattention and self-ratings of SBPS and QDQ showed a small to medium effect size. A simultaneous regression model was run on the whole group to predict SNAP-IV inattention score with age, gender, SBPS, and QDQ. The parent- and self- ratings of SBPS and QDQ were entered as predictors separately (see **Table 3**). Regarding the parent-ratings of SBPS and QDQ, the regression appeared fairly linear and homoscedastic as assessed by plots of the standardized residuals against the standardized predicted values. The Durbin–Watson statistic was 1.87, indicating that the residuals were independent. There was no evidence of multicollinearity, as assessed by the variance inflation factor (VIF= 1.04 – 1.80) and the tolerance (.56 -.96). The assumption of normality was met, as assessed by a visual inspection of the Q–Q plots. The results of the regression model indicated that both parent-ratings of SBPS and QDQ were significant and unique predictors of inattentive behaviors. The overall model predicted 53% of variance (*F* (4, 178 = 50.82, *p* <.001). The regression model of self- ratings of SBPS and QDQ predicting inattentive behaviors appeared fairly linear and homoscedastic, as assessed by plots of the standardized residuals against the standardized predicted values. The Durbin–Watson statistic was 1.30. There was no evidence of multicollinearity as assessed by the variance inflation factor (VIF = 1.01 – 1.22) and the tolerance (.82 -.99). The assumption of normality was met as assessed by a visual inspection of the Q–Q plots. The results of the regression model indicated that only the QDQ was a significant predictor of inattentive behaviors. The overall model predicted 10% of variance (*F* (4, 178) = 6.55, *p* <.001).

**Table 2:** Intercorrelations among parent and self-ratings of inattention symptoms, boredom proneness, and delay aversion, controlling for age and gender (*N* = 183).

Total score	SNAP-Inatt_P	SBPS_P	QDQ_P	SBPS_S	QDQ_S
**SNAP_Inatt_P**	–	.70***	.59***	.20**	.32***
**SBPS_P**		–	.64***	.16*	.20**
**QDQ_P**			–	.16*	.34***
**SBPS_S**				–	.41***
**QDQ_S**					–
**Mean**	11.24	24.40	29.55	22.73	27.40
**SD**	6.58	7.50	7.52	6.28	7.22

SBPS, Short Boredom Proneness Scale; SNAP-Inatt, Swanson, Nolan, and Pelham, version IV, Inattention Scale; QDQ, Quick Delay Questionnaire; _P, parent-rating; _S, self-rating.

*p <.05; **p <.01; ***p <.001.

**Table 3:** Summary of multiple regression analysis.

Variable	*B*	SE*_B_*	*β*	*t*	Sig.	95% CI
Dependent variable: SNAP-Inatt_P						
**Intercept**	-4.20	2.95		-1.43	.16	(-10.01, 1.62)
**Age**	-.21	.18	-.06	-1.15	.25	(-.57,.15)
**Gender**	-.05	.95	-.003	-.05	.96	(-1.93, 1.83)
**SBPS_P**	.48	.06	.54	7.99	<.001	(.36,.59)
**QDQ_P**	.22	.06	.25	3.61	<.001	(.10,.34)
***R* = .73, adjusted *R* ^2^ = .53, *F* (4, 178) = 50.82, *p* <.001**						
Dependent variable: SNAP-Inatt_P						
**Intercept**	3.64	3.91		.93	.35	(-4.07, 11.35)
**Age**	-.25	.24	-.07	-1.04	.30	(-.72,.22)
**Gender**	1.94	1.28	.11	1.51	.13	(-.59, 4.47)
**SBPS_S**	.09	.08	.08	1.09	.28	(-.07,.25)
**QDQ_S**	.26	.07	.28	3.63	<.001	(.12,.39)
***R* = .36, adjusted *R* ^2^ = .11, *F* (4, 178) = 6.55, *p* <.001**						

*B*, unstandardized coefficient; *β*, standardized coefficient; SE*_B_*, standard error of the coefficient; SBPS, Short Boredom Proneness Scale; SNAP-Inatt, Swanson, Nolan, and Pelham, version IV, Inattention Scale; QDQ, Quick Delay Questionnaire; _P, parent-rating; _S, self-rating.

### 3.3 The intermediary effect of delay aversion on the relation between boredom proneness and inattentive behavior

**Figure 1:**
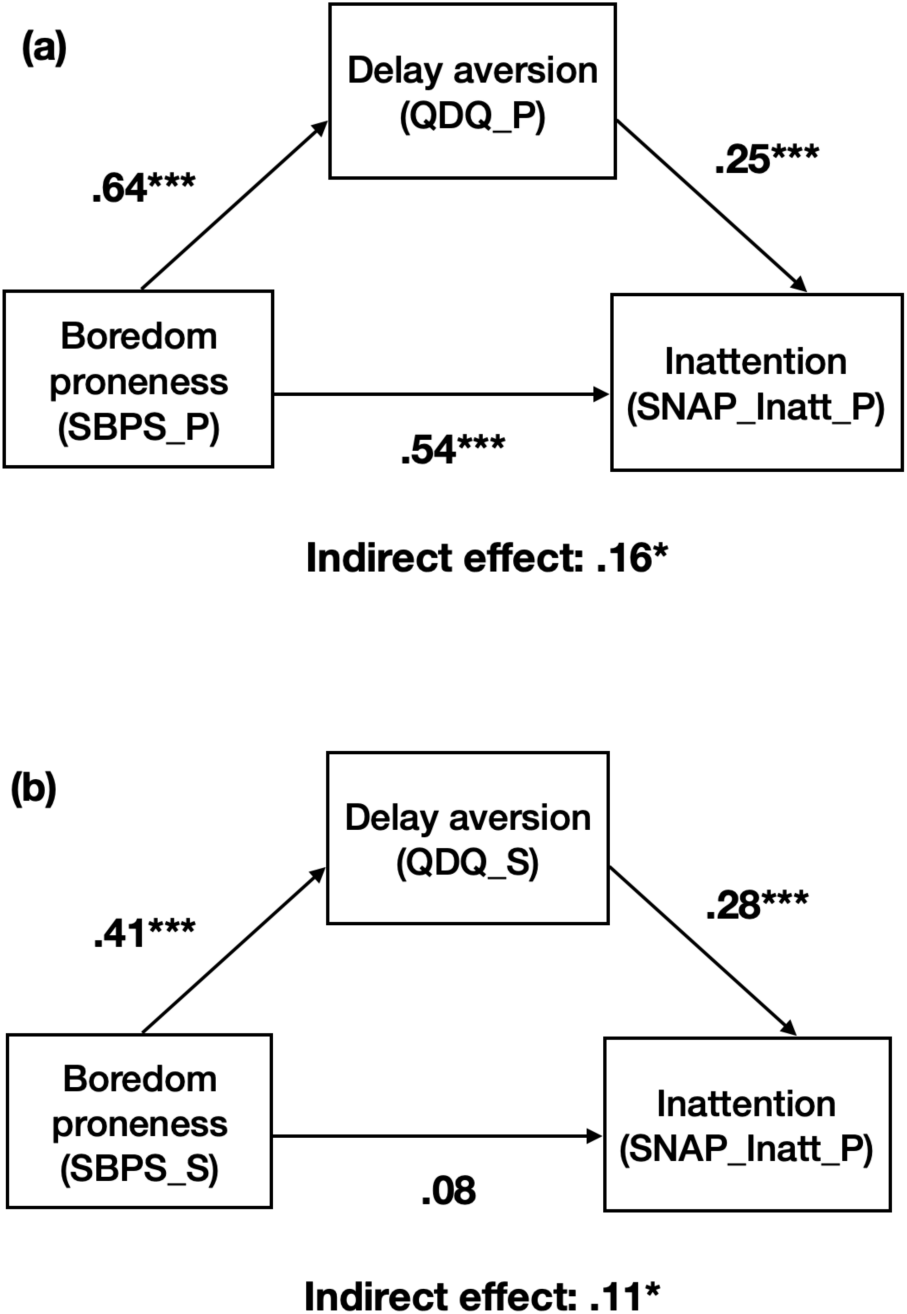
Intermediary effects of delay aversion on the relation between boredom proneness and inattentive behaviors. **(A)** Parent-rating of QDQ on the relation between parent-rating SBPS and parent-rating of SNAP-IV, **(B)** self-rating of QDQ on the relation between self-rating SBPS and parent-rating of SNAP-IV. SBPS, Short Boredom Proneness Scale; SNAP-Inatt, Swanson, Nolan, and Pelham, version IV, Inattention Scale; QDQ, Quick Delay Questionnaire. _P represents parent-rating; _S represents self-rating. Standardized *β* was reported. * p < .05, *** p < .001.

Summarized in **Table 4** and **Figure 1** are the results of the mediation models for the intermediary effects of QDQ on the relation between SBPS scores and inattention severity for the whole sample, with age and gender as covariates. The mediation analysis was conducted using the whole sample, as a separate analysis for the ADHD group and control groups yielded similar results. To simplify the results, we combined the data and present the analysis using the entire sample. The parent-ratings and self-ratings of QDQ and SBPS were modeled separately.

**Table 4:** Mediating effect of delay aversion between boredom proneness and inattention, with age and gender as covariates.

	Coefficient/effect	Standardized coefficient	SE	*t*	*p*	95% CI
**Model: 1**	Mediator: QDQ_P, covariates: age, gender					
	SBPS_P ➔ SNAP-IV_Inatt_P					
**A**	.64	.64	.06	11.21	<.001	(.53,.75)
**B**	.22	.25	.06	3.61	<.001	(.10,.34)
**c’**	.48	.54	.06	7.99	<.001	(.36,.59)
**C**	.61	.70	.05	13.02	<.001	(.52,.71)
**Indirect effect**	.14	.16	.04			(.05,.23)
**Model**	*R* = .71, *R* ^2^ = .50, *F* (3, 179) = 59.43, *p* <.001					
**Model: 2**	Mediator: QDQ_S, Covatiates: Age, gender					
	SBPS_S ➔ SNAP-IV_Inatt_P					
**A**	.47	.41	.08	6.05	<.001	(.32,.62)
**B**	.26	.28	.07	3.63	<.001	(.12,.40)
**c’**	.09	.08	.09	1.09	.28	(-.07,.25)
**C**	.21	.20	.08	2.74	.007	(.06,.36)
**Indirect effect**	.12	.11	.04			(.04,.20)
**Model**	*R* = .25, *R* ^2^ = .06, *F* (3, 179) = 4.07, *p* = .008					

Model 1: The mediation analysis of parent-rating of delay aversion on parent-ratings of boredom proneness and inattentive symptoms. Model 2: The mediation analysis of self-rating of delay aversion on self-rating of boredom proneness and parent-rating of inattentive symptoms.

SBPS, Short Boredom Proneness Scale; SNAP-Inatt, Swanson, Nolan, and Pelham, version IV, Inattention Scale; QDQ, Quick Delay Questionnaire; _P, parent-rating; _S, self-rating.

As to the model of parent-ratings, the overall model effect was statistically significant (*R = .*71, adjusted *R* ^2^ = .50, *F* (3, 179) = 59.43, *p* <.001), with a standardized total effect of.70, 95% CI:.52 to.71, *p* <.001. There was a significant direct effect of parent-rating SBPS score on inattentive behaviors (c’ = .48, *p* <.001, 95% CI:.36 to.60). The parent-rating of SBPS predicted the parent-rating QDQ, which, in turn, predicted the inattentive behaviors (all *p* <.001). The effect of parent-rating SBPS on inattentive behaviors was significantly mediated by parent-rating QDQ (the indirect effect:.14, 95% CI:.05 to.23). Age was a significant predictor in the relationship between SBPS and QDQ (*B* =.87, t(179)= -3.97, *p* <.001) and in the relationship between SBPS and SNAP (*B* = -.40, t(179)= -2.20, *p* <.001). The effects of age on the relationships between QDQ and SNAP were insignificant. The effects of gender were insignificant.

Regarding the model of self-ratings, the overall model was statistically significant (*R = .*25, adjusted *R* ^2^ = .06, *F* (3, 179) = 4.07, *p* = .008), with a standardized total effect of.21, 95% CI:.06 to.36, *p* = .007. The direct effect of self-rating SBPS score on inattentive behaviors failed to reach statistical significance (c’ = .09, *p* =.28, 95% CI: -.07 to.25). The self-rating of SBPS predicted the self-rating QDQ, which, in turn, predicted the inattentive behaviors (all *p* <.001). The effect of self-rating SBPS on inattentive behaviors was significantly mediated by self-rating QDQ (the indirect effect:.12, 95% CI:.04 to.21).The effects of age and gender on the relationships between SBPS and SNAP, QDQ and SNAP, and SBPS and SNAP were all insignificant.

## 4 Discussion

This study examined the associations among boredom proneness, delay aversion, and ADHD inattentive behaviors in children with and without ADHD. The ADHD group displayed significantly higher levels of boredom and delay aversion compared to the controls. Based on parent-ratings, boredom proneness and delay aversion were positively correlated and were both significant and unique predictors of ADHD inattentive behaviors. Delay aversion mediated the relation between boredom proneness and inattention. In terms of self-ratings, the psychometric properties of self-ratings of boredom proneness and delay aversion were not as good as those of parent-ratings. We found that self-ratings of boredom and delay aversion positively correlated to parent-rating of inattention, but self-rating of boredom proneness was not a significant predictor of parent-ratings of inattention. In general, the results suggest that boredom and delay aversion share variance in the prediction of ADHD-like inattentive behaviors.

This study is the first to investigate the correlations among boredom proneness, delay aversion, and ADHD-related behaviors using both parent- and self-ratings. Our findings fit with the existing literature that children with ADHD differ from typically developing controls with regard to caregivers’ and self-reports of trait boredom and delay-related behaviors. The ability of boredom proneness and delay aversion to predict ADHD inattentive behaviors warrants further discussion.

### 4.1 Boredom and ADHD symptomatology

Our results add to the literature demonstrating the vulnerability of children with ADHD to boredom. Individuals prone to boredom make more errors in tasks that require sustained attention (9, 21). Specifically, Hunter and Eastwood (21) examined whether state boredom was the cause or consequence of attentional failures. The authors found that the levels of state boredom before and after blocks of the sustained attention task were positively correlated with performance errors, suggesting that boredom can be both the cause and a consequence of attentional failures. The current study examined a path from boredom proneness to inattentive behaviors. However, the two constructs were highly intercorrelated, and a bi-directional relationship may be possible.

### 4.2 Mediating effect of delay aversion

In this study, we examined the hypothesis that delay aversion mediates the relation between boredom proneness and ADHD symptom severity because the delay aversion hypothesis suggests that people with high levels of delay aversion experience negative feelings and attentional failures during delay. Moreover, the theoretical model of boredom proposes that it is closely related to attention failure. Our data on parent-ratings demonstrated that the link between boredom and ADHD inattentive behaviors was mediated by delay aversion. Interestingly, children’s self-perceived boredom proneness failed to predict parent-assessed inattentive behaviors, although children with ADHD reported a significantly higher level of boredom proneness than children without ADHD. The discrepancy between child self-ratings and parent-ratings commonly exists in children with ADHD (34). Children’s self-evaluations of their functioning tend to be more positive than parents’ evaluation in general. We suggest that parent-rating is a more sensitive measure of the relations among boredom proneness, delay aversion, and inattentive behaviors than self-ratings.

Although this study focused on propensity measures, the findings were in line with those of Wilbertz et al. (25), who reported that adults with ADHD displayed significantly increased levels of state boredom, impatience, and negative affect during delay compared with healthy controls. Moreover, activity in the right amygdala increased with longer delays and was correlated with the degree of behavioral aversion to delay in the ADHD group, whereas amydgala activity decreased with the delay length in the control group. The study by Wilbertz et al. (25) is important because it is one of the few studies to experimentally measure the state of boredom in individuals with ADHD despite a small sample size. Future studies investigating the in the moment emotional and behavioral consequences of boredom and delay aversion with larger sample sizes of individuals with ADHD are needed.

### 4.3 Mechanisms underlying boredom, delay aversion, and ADHD

The results of the current study suggest that boredom plays a role in delay aversion and ADHD symptoms. Boredom is closely linked to regions of the brain’s default mode network (35). Research has found that boredom-induced negative affect was associated with increased activities in the bilateral ventromedial prefrontal cortex and decreased activity in the precuneus (36). Ulrich et al. (37) examined the neural substrates of boredom (when the task demand was low), flow (when the task demand was adjusted to the personal skill level), and overload conditions when the participants underwent perfusion MRI. The authors reported increased cerebral blood flow in the MPFC and left amygdala, hippocampus, and parahippocampal gyrus during the boredom condition compared to flow and overload conditions. Danckert and Merrifield demonstrated that resting state default mode network activity closely overlapped those observed during two experimentally manipulated boredom conditions (38). Notably, there was a negative correlation between the DMN and insula during the two boredom conditions. As the insula performs a critical role in modulating the integration of externally oriented processing and internal states (39, 40), the authors have suggested that boredom may be associated with switching between internal mentation and external information processing.

DMN’s role in boredom also connects to ADHD. Excessive DMN activity has been linked to attention lapses, a common feature of ADHD (41–43). Individuals with ADHD exhibit abnormalities in DMN functional connectivity (44, 45) and struggle to transition from resting to external goal-directed tasks (16, 46). An EGG study further reveals that children with ADHD show reduced attenuation of very low frequency (VLF) EEG activity within DMN areas when transitioning from rest to tasks or waiting, with reduced attenuation correlating with parent-rating of delay aversion (47). These findings suggest that boredom proneness and delay aversion are interconnected and contribute to ADHD symptoms.

### 4.4 Implication and intervention

The current study suggests that individuals who are more prone to boredom are also more averse to delay, which, in turn, increases the likelihood of inattentive behaviors in both children with and without ADHD. If children learn better strategies to cope with the boredom during periods of delay, they can engage in more enriching activities and sustain their attention. Play is a natural gift that helps children navigate challenges and stress. A recent study on toddlers found that the ability to adapt and shift play styles helps reduce boredom and affects how long they remain engaged in play (48). Helping children discover enjoyment in daily activities, encouraging their imagination and creativity, may alleviate boredom and inattention. Furthermore, parental responsiveness appears to be linked to boredom and ADHD-like behaviors (49), suggesting that parent–child interaction plays a crucial role in shaping children’s ability to regulate boredom and maintain attention.

### 4.5 Limitations of the study

This study has some limitations. First, the reliance of our data on parent and self-reports may introduce subjective biases. Second, the results were based on cross-sectional data, and one must be highly cautious about drawing causal inferences. A study design with longitudinal follow-up or experimental manipulations will test the causal inferences suggested here. Third, our study focused on the intermediary role of delay aversion on the relation between boredom and inattention. However, the relations between boredom trait and delay aversion inattention may be bi-directional. Future studies could comprehensively explore this relationship through the application of path analysis.

## 5 Conclusion

Boredom proneness and delay aversion in ADHD have tended to be investigated independently in the literature. Our study found that boredom proneness and delay aversion were closely linked and significantly predicted ADHD inattentive behaviors. Investigating the impact of boredom and delay aversion will facilitate better life functioning for individuals with ADHD.

## Data Availability

The original contributions presented in the study are included in the article/supplementary material. Further inquiries can be directed to the corresponding author.
